# Peripapillary Oxygenation and Retinal Vascular Responsiveness to Flicker Light in Primary Open Angle Glaucoma

**DOI:** 10.3390/metabo12070597

**Published:** 2022-06-27

**Authors:** Cengiz Türksever, Margarita G. Todorova

**Affiliations:** 1 VISTA Clinic Binningen, CH-4102 Binningen, Switzerland; 2 Department of Ophthalmology, Cantonal Hospital St. Gallen, CH-9007 St. Gallen, Switzerland; margarita.todorova@kssg.ch; 3 Department of Ophthalmology, University of Zürich, CH-8032 Zürich, Switzerland; 4 Department of Ophthalmology, University of Basel, CH-4001 Basel, Switzerland

**Keywords:** ocular blood flow, glaucoma, optic nerve damage, retinal vessel oxygen saturation, parapapillary oxygen exposure, retinal vascular responsiveness to flicker light

## Abstract

The aim of our study was to evaluate peripapillary oxygenation and its relationship to retinal vascular responsiveness to flicker light in patients with primary open angle glaucoma (POAG). Retinal vessel oxygen saturation was measured in 46 eyes of 34 Caucasian patients with POAG and in 21 eyes of 17 age-matched controls using the oximetry tool of Retinal Vessel Analyser (RVA: IMEDOS Systems UG, Jena, Germany). The mean oxygen saturation of the major arterioles (A-SO_2_; %) and venules (V-SO_2_; %), as well as the corresponding arterio–venular difference (A-V SO_2_; %), were calculated. We also measured retinal vascular responsiveness (RVR) to flicker light by means of RVA. Glaucoma patients were divided in two subgroups according to their median arteriolar and venular vascular responsiveness to flicker light (AFR and VFR). Glaucomatous damage was assessed by optical coherence tomography (Carl Zeiss Meditec, Dublin, CA, USA) and static automated perimetry (Octopus, program G2/standard strategy: Haag-Streit International, Köniz, Switzerland). In addition, we calculated the mean peripapillary oxygen exposure [ppO_2_E; %/µm] by dividing the mean A-V SO_2_ with the mean retinal nerve fibre layer (RNFL) thickness. In glaucoma patients, A-SO_2_ and V-SO_2_ values were significantly increased, and their difference decreased when compared to controls (*p* < 0.017; linear mixed-effects model). Grouped with respect to retinal vascular responsiveness to flicker light, subjects with reduced VFR (≤2.9%) had significantly higher ppO_2_E (0.49 ± 0.08%/µm, respectively, 0.43 ± 0.06%/µm; *p* = 0.027). Additionally, higher ppO_2_E in glaucoma patients correlated negatively with the neuroretinal rim area (*p* < 0.001) and the RNFL thickness (*p* = 0.017), and positively with the mean defect of the visual field (*p* = 0.012). Reduced venular vascular responsiveness in our glaucoma patients was associated with increased peripapillary oxygenation exposure. Thus, ganglion cells and their axons in glaucomatous eyes with reduced retinal vascular responsiveness are prone to be more exposed to higher oxidative stress, probably contributing to the further progression of glaucomatous damage.

## 1. Introduction

Glaucoma is a chronic optic neuropathy leading to progressive visual field (VF) defects and corresponding morphological alterations of the optic nerve head. Increased intraocular pressure (IOP) has long been proposed as a major risk factor for primary open angle glaucoma (POAG). 

However, aside from increased IOP, retinal- and choroidal blood flow alterations are discussed as important players in the pathogenesis of glaucoma [1,2]. A variety of hemodynamic studies using laser Doppler velocimetry (LDF), colour Doppler imaging and retinal functional imaging revealed reduced blood flow of the iridal, retinal, choroidal and retrobulbar vasculature in glaucoma patients [2,3,4,5,6,7,8,9,10]. Furthermore, the reduced choroidal blood flow correlated positively with the central retinal dysfunction, measured by the use of pattern ERG [11]. 

In general, the sufficient regulation of ocular blood flow compensates for instability in perfusion pressure (autoregulation) and adapts to retinal activity (neurovascular coupling) [2,9,12]. Whereas the retinal vessels are influenced by the activity of the neural and glial cells, the choroidal and the optic disc vessels are influenced further by the activity of circulating vasoactive molecules, such as Endothelin-1 and angiotensin II. Due to its direct access to the smooth muscle cells and pericytes of the vessels, these circulating molecules influence the blood–retinal barrier and thus the regulation of choroidal an optic nerve blood flow [2,9,12]. 

Meanwhile, insufficient blood supply is supposed as a confounding factor for glaucoma progression [2,10,12,13,14,15,16]. According to these data, when the ocular blood flow is reduced, the optic nerve becomes more sensitive to increase in IOP but also to decreases in perfusion pressure. Noticeably, perfusion instability, rather than a steady reduction in ocular blood flow, is thought to contribute to glaucomatous damage [14]. Insufficient autoregulation increases further the chance for the unstable ocular perfusion of unstable oxygen supply, which is no longer proportional to oxygen demand, leading thereby to increase in oxygen free radicals and oxidative stress [17]. 

Due to vascular dysregulation in glaucoma, the oxygen supply of the optic nerve is not sufficiently regulated upon oxygen demand of the inner retina. All of the above leads in turn to the further progression of glaucomatous optic neuropathy [2,12,15]. Thus, glaucoma today entails a complex interaction between retinal blood flow, choroidal blood flow, oxygen delivery, oxygen consumption and oxygen exposure. 

Oxygen is known to be the most supplied metabolite in the retina, and the regulation of adequate oxygen supply and cellular energy metabolism is required to maintain a healthy retinal function [18,19]. This regulation is especially critical to the ganglion cells and their axons, as for instance in glaucoma, where energy consumption is highly dynamic.

Spectrophotometric retinal vessel oximetry (RO) provides a non-invasive measurement of oxygen saturation in retinal vessels [20]. A review of the literature suggests the retinal oxygen metabolism to be involved in the pathogenesis of glaucoma. Recently, in glaucoma, an altered oxygen metabolism and its association with structural and functional parameters was reported [21,22,23,24,25,26]. Most of the cited studies confirmed higher oxygen saturation in the venules (V-SO_2_) of glaucoma patients but also a lower arteriolar–venular difference (A-V SO_2_), the latter known to be proportional to the oxygen use. These findings were more pronounced in patients with advanced glaucoma. For instance, the A-V SO_2_ has been found to correlate negatively with the VF defect and positively with the neuroretinal rim area and with the peripapillary retinal nerve fibre layer (RNFL) thickness [21,22,23,24,25]. This observation has been thought to be related to the reduced metabolism, resulting from tissue loss in glaucoma. Interestingly, compared to patients with POWG, in normal tension glaucoma (NTG) patients, a reduced A-SO_2_ has been found as well [21,25]. The latter has been explained as related to reduced ocular blood flow and, thus, to reduced oxygen delivery [25]. However, the amount of retinal tissue is altered with a corresponding loss of nerve fibre layer and ganglion cells. Accordingly, reduced neuronal tissue needs less oxygen, which in glaucomatous eyes is not properly regulated. 

Together, these results indicated the crucial role of oxygen metabolism in the pathogenesis of glaucoma and even more so in eyes featuring ocular blood flow alteration. Thus, the primary aim of the present study is to evaluate peripapillary oxygenation and its relationship to retinal vascular responsiveness to flicker light in POAG. Although the correlation between the level of glaucomatous damage and retinal vessel oxygen saturation has been confirmed in previous studies, peripapillary oxygen exposure for a certain amount of RNFL tissue [ppO_2_E; %/µm] has not yet been studied. In order to accomplish this, we calculated the mean retinal peripapillary oxygen exposure per micron of nerve fibre layer thickness, ppO_2_E [%/µm], by dividing the mean A-V SO_2_ by the mean RNFL thickness. The second aim of the present study is to evaluate the relationship between the peripapillary retinal oxygen exposure in glaucoma patients and the neuro–vascular coupling as measured by retinal vascular responsiveness to flicker light in POAG. 

## 2. Results

The study adhered to the tenets of the Declaration of Helsinki. The study was approved by local authorities (Ethics Commission of Central and Northern Switzerland, EKNZ Basel Switzerland) with a positive vote for observational investigations (trial number EKNZ BASEC 2017-00937). Participants were enrolled based on the criteria described in the Section 4. 

A total of 46 eyes of 34 selected Caucasian (18 female and 16 male; mean age: 65.52 (SD: ±14.84)) patients with open angle glaucoma and twenty-one eyes of 17 (8 female and 19 male) age-matched controls (mean age: 63.21(SD: ±7.65); *p* = 0.523), suffering no ophthalmic pathology, were enrolled in the study. 

All glaucoma patients had a glaucomatous optic neuropathy with a mean optic disc area of 0.87 mm^2^ (±0.29) and a mean peripapillary RNFL thickness of 78.37 µm (±14.80). The mean visual field (VF) defect (MD) (Octopus perimetry; G2 standard program) was 4.91 dB (±5.46). Among all glaucoma patients, the mean IOP at the time of examination was below 21 mmHg: (mean 15.85 mmHg; ±3.15). 

With regard to therapy for our glaucoma patients’ eyes: 17 had prostaglandins, 10 beta-blockers, 2 diuretics, and 15 combined IOP lowering medication (31 eyes had more than one of the mentioned medications). The glaucoma surgical procedures on our glaucoma patients’ eyes consisted of eight trabeculectomy procedures with Mitomycin and 21 selective laser trabeculoplasty procedures. Twenty-three eyes were administered a combination of glaucoma medication and surgery.

### 2.1. Oxygen Saturation Parameters in Glaucoma vs. Controls

In glaucoma patients, the arteriolar (A-SO_2_; %) and venular oxygen saturation (V-SO_2_; %) values were significantly increased and their difference (A-V SO_2_; %) decreased when compared to controls (ANOVA based on linear mixed-effects model; Table 1). For instance, the mean (±SD) A-SO_2_, V-SO_2_ and A-V SO_2_ (%) in glaucoma patients were: 96.18% (±3.64), 61.66% (±6.04) and 34.86% (±6.04), whereas in controls, respectively: 93.44% (±4.97), 54.21% (±9.10) and 39.25% (±7.96) (*p* = 0.016; *p* < 0.001, and *p* = 0.011). Peripapillary oxygen exposure (ppO_2_E; %/µm) in POAG patients was significantly increased compared to controls. For instance, the corresponding mean ppO_2_E (±SD) values for the glaucoma group were 0.46%/µm (±0.10) and for the age-matched controls: 0.40%/µm (±0.09; *p* = 0.024; Table 1). 

### 2.2. Oxygen Saturation by Vascular Responsiveness in Glaucoma Patients

When the venular flicker responsiveness (VFR) within the glaucoma patients’ group was taken into account (≤/>2.9%), neither the A–SO_2_, the V–SO_2_ nor the A–V SO_2_ showed a statistically significant difference between the glaucoma VFR subgroups (*p* = 0.517; *p* = 0.391 and *p* = 0.294, respectively; Table 2). The same held true for the vascular responsiveness of the arterial side (AFR) (≤/>1.80%): here none of the following parameters showed statistically significant difference between AFR groups; A–SO_2_, V-SO_2_, A–V SO_2_ and ppO_2_E (*p* > 0.082).

The ppO_2_E was, however, higher in the glaucoma subgroup with reduced VFR (≤2.9). The respective mean (±SD) values of the ppO_2_E within the VFR≤ 2.9 group were 0.49%/µm; (±0.08) and within the VFR < 2.90 group were 0.43%/µm; (±0.06) (*p* = 0.027, Table 2).

### 2.3. Oxygen Saturation and Glaucomatous Damage

Within our glaucoma group, the relationship between the oxygen saturation measurements and the extent of glaucomatous damage was evidenced by statistically significant values. Interactions were calculated based on linear mixed-effects model. Categorical predictors are presented as differences of means with corresponding 95% C.I. and *p*-values. For continuous predictors, results are expressed as differences of means (slope) increasing the predictor by one unit. 

Both the V-SO_2_ and the A-V SO_2_ correlated with the extent of glaucomatous damage, as measured by the neuroretinal rim area, the peripapillary RNFL thickness and the mean MD of the visual field. Here, increased V-SO_2_ and decreased A-V SO_2_ in our glaucoma patients correlated positively with smaller rim area, thinner RNFL and deeper visual field defects (*p* ≤ 0.012, Table 3). For instance, for the V-SO_2_ the interactions were the following: neuroretinal rim area (*p* = 0.012; slope: −9.160 [CI: −14.830–−3.490]; %/mm^2^), RNFL thickness (*p* = 0.001; slope: −0.161 [CI: −0.280–−0.042]; %/µm) and VF (*p* < 0.002; slope: 0.452 [CI: 0.090–0.814]; %/dB). For the A-V SO_2_, the corresponding interactions were the following: neuroretinal rim area (*p* = 0.007; slope: 5.962 [CI: 0.656–11.269]; %/mm^2^), RNFL thickness (*p* < 0.001; slope: 0.120 [CI: 0.014–0.225]; %/µm) and VF (*p* = 0.001; slope: −0.306 [CI: −0.603–−0.010]; %/dB; Table 3). 

The A-SO_2_ showed no significant relationship with the psychophysical and structural measures in our glaucoma patients. 

### 2.4. Peripapillary Oxygen Exposure and Glaucomatous Damage

In regard to the ppO_2_E and its relation to the extent of glaucomatous damage, higher ppO_2_E in glaucoma patients correlated negatively with the neuroretinal rim area (*p* < 0.001) and the RNFL thickness (*p* = 0.017) but positively with the mean defect of the VF (*p* = 0.011; Table 3). The corresponding interactions of ppO_2_E with the extent of glaucomatous damage were the following: neuroretinal rim area (*p* < 0.001; slope: −0.004 [CI: −0.006–−0.003]), %/µm/mm^2^); RNFL thickness (*p* = 0.017; slope: −1.609 [CI: −0.249–−0.073], %/µm/µm,) and VF (*p* = 0.007; slope: 24.570 [CI: 0.002–0.012], %/µm/dB; Table 3).

### 2.5. Vascular Flicker Light Responsiveness vs. Glaucomatous Damage

When the vascular responsiveness was taken into account, glaucoma patients with lower venular vascular responsiveness (VFR ≤ 2.9%) showed significantly thinner RNFL (*p* = 0.014), borderline smaller rim area (*p* = 0.098) and slightly deeper visual field defects (*p* = 0.270) when compared to glaucoma patients with higher venular vascular responsiveness (Table 4). 

### 2.6. Oxygen Saturation vs. Peripapillary Oxygen Exposure

Glaucoma patients with increased ppO_2_E (>0.46%/µm) had significantly higher V-SO_2_ values when compared to controls (*p* = 0.015) and showed a trend for V-SO_2_ to increase when compared to glaucoma patients with reduced ppO_2_E (*p* = 0.053). The A-V SO_2_ was significantly reduced in glaucoma patients with increased ppO_2_E (>0.46%/µm), compared to glaucoma patients with reduced ppO_2_E (≤0.46%/µm; *p* = 0.008), and showed a trend for A-V SO_2_ to decrease when compared to controls (*p* = 0.060; Table 5). 

## 3. Discussion

In the current study, we analysed oxygen metabolism and peripapillary oxygen exposure of the inner retina as well as their relation to retinal vascular responsiveness to flicker light and glaucomatous damage in glaucoma patients. Alteration in oxygen metabolism has been discussed in the pathologic chain of events in glaucoma patients [21,22,23,24,25,27]. Most of the cited studies confirmed higher oxygen saturation in the venules (V-SO_2_) of glaucoma patients but also a lower arteriolar–venular difference (A-V SO_2_), the latter known to be proportional to the oxygen use. These findings were more pronounced in patients with advanced glaucoma. We confirmed the altered metabolic function in glaucoma, showing, however, a slightly different metabolic pattern. Compared to the studies sited above, our glaucoma patients revealed increased V-SO_2_ but instead also decreased A-V SO_2_ (*p* < 0.017) when compared to controls. This result may partly be influenced by the assignment of the method applied and the cohort of glaucoma patients recruited in the studies. However, the result could also depend on retinal vascular responsiveness. 

As Graham supposed previously, the oxygen consumption is a product of arterio–venular difference (A-V SO_2_) and retinal blood flow [28]. Therefore, choroidal circulation supplying the outer retina indirectly effects the inner retina. However, in terms of oxygen consumption, not only choroidal and retinal blood flow but also oxygen delivery is an important factor. In case of sufficient oxygen supply, the oxygen consumption should be independent of the ocular blood flow [29]. Additionally, altered neurovascular coupling in glaucoma patients has been discussed as related to vascular dysregulation as measured by flicker stimulation [27,30,31,32].

Therefore, in order to evaluate objectively the effect of vascular dysregulation on metabolic alterations in glaucoma, we subdivided our glaucoma patients according to their median vascular flicker light responsiveness. 

### 3.1. Oxygen Saturation and Glaucomatous Damage

Previous research on retinal vessel oxygen saturation has proposed a link between the metabolic and the structure–functional alterations in glaucoma patients [21,22,23,24]. In agreement with these findings, we confirm again a clear relation between the level of glaucomatous damage and the degree of metabolic dysfunction: V-SO_2_ became higher and A-V SO_2_ became lower with the progression of glaucomatous damage, as documented by measuring the neuronal rim area, the RNFL thickness and the visual fields (Table 4). Our results could be explained by reduced ocular blood flow and decreased perfusion pressure of the optic nerve, both of which have already been discussed as players in the pathogenesis of glaucoma progression [2,12,15]. 

It has been hypothesized that altered ocular perfusion and unstable oxygen supply lead to the increased sensitivity of the optic nerve head to an increase in IOP but also to increase in oxygen free radicals and oxidative stress in the intercellular space [17]. We suppose, therefore, that the oxygen saturation alterations in glaucoma, as discussed in previous studies [21,22,23,24] and also measured in the present study, in part result from reduced oxygen demand following glaucomatous axonal loss. All of the discussed above may led to the further progression of glaucomatous optic neuropathy. Exemplified in our study, an increase in V-SO_2_ and a decrease in A-V SO_2_ were linked to the degree of RNFL thinning and visual field loss. 

### 3.2. Vascular Responsiveness to Flicker Light Exposure in Glaucoma

Until now, RVA has been used to assess the vascular dysregulation pattern in patients with glaucoma but also in patients with early cardiovascular insufficiency or cerebrovascular disease [30,32,33,34]. In controls, under flicker light exposure an increase in retinal vessel diameter and retinal- and optic nerve head blood flow have been measured [27,34,35]. In POAG patients, when compared to age-matched controls, retinal vascular response to repeated flicker light stimulation has been found to be reduced and demonstrated a predominantly affected VFR pattern [27,30,31,32]. Reduced vascular responsiveness to flicker light is, however, a finding also observed in subjects with primarily vascular dysregulation [32]. As the blood supply from the short-posterior ciliary arteries has also been measured to be altered [9,36,37], an underlying vascular dysregulation in the pathogenesis of glaucoma has been hypothesized [38]. Therefore, in the presence of reduced vascular responsiveness to flicker light stimulation, more pronounced glaucomatous damage and, hence less perfusion demand were expected to be found. Interestingly, even confirming reduced vascular flicker light responsiveness on the arterial, as well as on the venous side in glaucoma [27,30,31,32], no statistically significant correlations have been confirmed either with the VF defect or the RNFL in the cited studies [39].

Thus, a novel finding in the present study is the significant interactions found between the reduced VFR and increased venular oxygen saturation but also increased peripapillary oxygen exposure. Nevertheless, we do not propose that glaucoma patients have primarily venular dysfunction but confirm that the venous side is also affected. This finding stays in agreement with previous research on glaucoma patients, demonstrating increased intraluminal venous pressure and resistance [40]. Even if we did not measure the intraluminal venous pressure of our glaucoma patients, it seems plausible the observed attenuation in VFR to be a result of increased retinal venous pressure. This means that increased retinal venous pressure observed in glaucoma patients [40,41,42] might reduce the dilatation ability of retinal venules. Therefore, not only increased IOP but also increased resistance of the peripheral vessels [40,43] seems to result in the inadequate perfusion of the optic nerve in glaucoma patients.

Based on our data of vascular flicker responsiveness, we found more altered metabolic function in those glaucoma patients suffering reduced VFR. Furthermore, glaucoma patients with reduced venular vascular responsiveness (≤2.9%) showed higher peripapillary oxygen exposure (ppO_2_E; *p* = 0.027; Table 2). Additionally, among glaucoma patients, those with higher ppO_2_E showed significantly smaller rim area (*p* < 0.017), thinner RNFL (*p* < 0.001) and deeper visual field defects (*p* = 0.011) compared to glaucoma patients with lower ppO_2_E (Table 3). A possible explanation of the discussed above could be the fact reported in the literature that in case of sufficient neurovascular coupling the flicker light, stimulation would result in increase in retinal blood flow [27,30]. On contrary, in glaucoma patients with altered neurovascular coupling one would expect reduced responsiveness to flicker light stimulation, reduced retinal blood flow and altered oxygen metabolism, a finding we have now confirmed in the present study. All these alterations in terms of neurovascular coupling probably led to increased peripapillary oxygen exposure within the RNFL. 

### 3.3. Vascular Flicker Light Responsiveness vs. Oxygen Saturation in Glaucoma

Evaluating vascular arteriolar or venular flicker light responsiveness against retinal vessels oxygen parameters, we found no statistically significant correlations (*p* > 0.295). This means that the amount of flicker responsiveness in terms of vessel dilatation from baseline diameter of vessels did not correlate directly with the loss of VF and RNFL thickness. This is, however, to be expected, as due to vascular dysregulation, a direct correlation of these parameters is not plausible. As result of vascular dysregulation, the oxygen in the inner retina is not proportionally delivered, now exemplified by significantly altered metabolic parameters, such as, for instance, the ppO_2_E and A-V SO_2_. Since the number of ganglion cells is reduced in glaucoma, less oxygen is necessary. However, the oxygen delivery due to vascular dysregulation remains, in some patients, proportionally high, as already discussed regarding oxidative stress-related disease progression in the pathogenesis of glaucoma [17]. 

Thus, again, our results confirm severely disturbed retinal vascular regulation ability to flicker light in glaucoma patients in general, even if slightly more disturbed in those suffering from vascular dysregulation [31,32,38]. 

Indeed, retinal vascular responsiveness and oxygenation are not only altered in patients with glaucoma but also in patients with other diseases, such as those with diabetic retinopathy as well as in retinitis pigmentosa [44,45,46,47]. However, all three entities show a different pattern and pathological background.

Interestingly, we found the RNFL thickness in glaucoma patients with reduced VFR to be significantly thinner than in those with normal VFR (*p* = 0.014, Table 4). The latter could be explained with the following: vascular dysregulation in glaucoma patients has been hypothesized to lead to the excessive oxygenation of the RNFL and to increased oxidative stress, causing further RNFL loss, thus contributing to further glaucomatous damage. We suppose, therefore, that in subjects featuring vascular dysregulation, the oxygen is not delivered to the ganglion cell axons in the inner retina, thus leading to decreased O_2_ demand. 

### 3.4. Peripapillary Oxygen Exposure in RNFL

Several studies made an effort to evaluate inner retinal metabolism in healthy subjects. The study by Palkovits et al. calculated the retinal oxygen extraction, evaluating inner retinal oxygen delivery and retinal blood flow using a combined retinal vessel oximetry/laser Doppler velocimetry approach [48]. This study revealed, in healthy subjects during 100% oxygen breathing, an increase in oxygen saturation in retinal arterioles and venules with a corresponding significant decrease in A-V SO_2_. The calculated oxygen extraction in the eye was found to decrease by 62.5 ± 9.5%. These findings suppose the oxygen extraction of healthy subjects to be well regulated and adaptive to different conditions, in contrast to glaucoma patients with vascular dysregulation. Hence, the high amount of oxygenation leads to oxidative stress and tissue damage in the retina.

Additionally, a direct relation between the level of glaucomatous damage and the retinal vessel oxygen saturation has been found in previous studies [21,22,23,24] and was confirmed in the present study as well. However, in the present study, we applied a different approach. Studying the inner retinal metabolism, we calculated the mean peripapillary oxygen exposure per micron of RNFL thickness, aiming indirectly to evaluate the effect of oxidative stress for a given amount of RNFL thickness. 

### 3.5. Peripapillary Oxygen Exposure and Vascular Dysregulation in Glaucoma

Another novel finding in the present study is the fact that the ppO_2_E values were significantly higher in glaucomatous eyes with reduced venular vascular responsiveness to flicker light. Our results clearly indicate that in glaucoma patients with vascular dysregulation, the ppO_2_E is proportionally higher than in patients with better vascular responsiveness to flicker light stimulation. Studying further the role of the peripapillary oxygen exposure on metabolic, structural and functional alterations in glaucoma patients, we found that glaucoma subjects with higher ppO_2_E revealed more affected neuroretinal rim area, reduced RNFL thickness and deeper visual field defects (*p* < 0.018). 

Not surprisingly, increased oxygen saturation, elevated oxygen metabolism and oxidative stress have been found in other neurodegenerative diseases, like Parkinson’s and Alzheimer’s disease. Interestingly, these findings were more pronounced in subjects featuring vascular dysregulation, compared to those with normal vascular responsiveness [49,50,51,52,53]. Indeed, similar to the neurodegenerative diseases, oxidative stress in glaucoma leads to cellular damage caused by reactive oxygen species. Increased oxidative stress results, in turn, in cytotoxicity, changes in the signalling pathway of retinal ganglion cells (RGS) death, in protein modification and glial dysfunction. In healthy subjects, there is a balance between the neutralization and production of oxygen free radicals. In glaucoma, oxygen-free radicals induce glial cells’ autoimmune reaction [54,55]. The extended oxygenation of RGC, due to reduced vascular regulation in terms of neurovascular coupling, triggers RGC death and results in the further progression of glaucoma [54,55]. In agreement with the previously stated, the dysfunction of the neurovascular coupling in glaucoma [31,56] may play a physiological role in regulating sensitivity and plasticity via oxygen depletion and the induction of downstream hypoxic response pathways, thus leading to an increase in oxygen free radicals and consequent peripapillary oxygen exposure, producing further glaucomatous damage.

### 3.6. Precautions

Identifying glaucoma patients with vascular dysregulation may allow undertaking preventive measures, such as targeting the eye pressure to lower values in order to improve the ocular perfusion pressure. A further attempt is the avoidance of increased oxygenation, while implementing preventive treatment with magnesium [57] and low dose calcium-channel blockers [58]. Since increased oxygen-free radicals seem to induce glial cells’ autoimmune reaction, resulting in further glaucomatous damage, the application of polyunsaturated fatty acids [59], Ginkgo biloba [60] and antioxidants [61] has been proposed. Nevertheless, their effect should be evaluated in further prospective studies. 

## 4. Materials and Methods

An observational pilot study was performed. Forty-six eyes of 34 Caucasian primary angle glaucoma patients (18 female and 16 male; age range: 34–84 y, mean 65.45 y; SD ± 11.7) were recruited through medical record review between 2012–2014 in the glaucoma unit of the Department of Ophthalmology at the University of Basel. Twenty-one eyes of 17 age-matched controls (8 female and 19 male; age range: 45–74 y, mean 63.21 y (SD: ±7.65); *p* = 0.523) suffering no ophthalmic pathology were evaluated as well.

We included patients with the characteristic pattern of the glaucomatous VF defect, corresponding to the glaucomatous alterations of the optic nerve head (based on cup/disc ratio, thinning of neuroretinal rim, notching and reduced retinal nerve fibre layer (RNFL) thickness disc haemorrhages, etc.). Glaucomatous VF defect was defined as repeatedly measured loss of 5 dB sensitivity in at least three contiguous points or a loss of 10 dB sensitivity in at least one point. 

All participants underwent standard ophthalmologic examination, including best-corrected visual acuity (BCVA, Snellen charts), Goldmann applanation tonometry measurement, biomicroscopy of the anterior segment and ophthalmoscopy of the posterior pole. 

Exclusion criteria for glaucoma patients’ participation in the study were: angle closure glaucoma, unclear optic media, unstable fixation and ocular and/or systemic pathology, which may influence the quality of retinal oximetry-, SD-OCT- and flicker light- (FL) response imaging. Exclusion criteria for controls’ participation in the study were: unclear optic media, unstable fixation and ocular and/or systemic pathology, which may influence the quality of retinal oximetry imaging and/or RVA analyses.

### 4.1. Visual Field Examination

Standard automated visual fields (VF) were obtained in all glaucoma patients for both eyes, starting with the right eye (G2 standard program, Octopus perimeter 101; Haag-Streit AG, Köniz, Switzerland). In all patients, the reliability factor of the VF examination was below 10.

### 4.2. Spectral Domain OCT

After pupillary dilatation using Tropicamide 0.5% and Phenylephrine 1% eye drops, a spectral domain optical coherence tomography (SD-OCT) imaging was obtained using the Cirrus HD OCT system (Cirrus HD-OCT; Carl Zeiss Meditec, Dublin, CA, USA). RNFL thickness measurements were performed using the optic disc cube mode [26]. Subjects with more than 6 signal strength were excluded from the study.

### 4.3. Retinal Vessel Oximetry and Retinal Vessel Analyser Imaging

**Figure 1:**
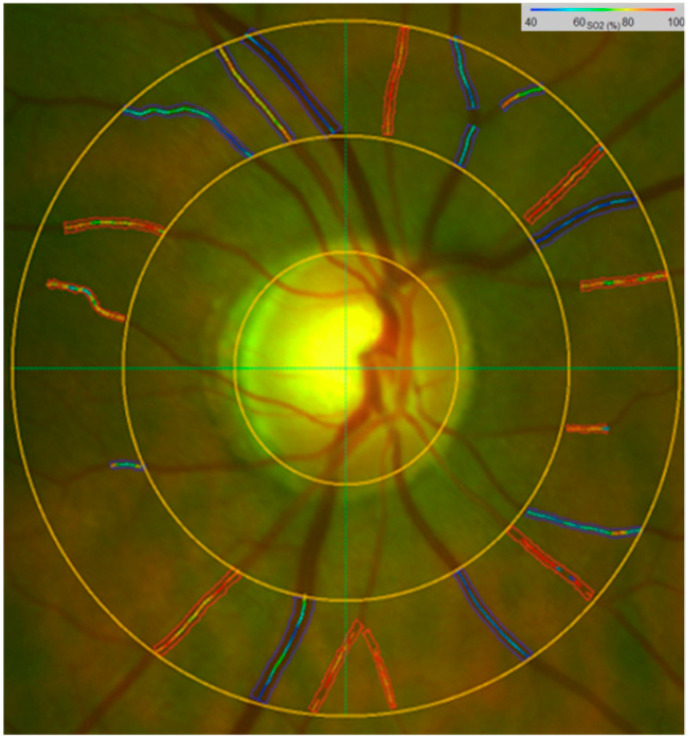
A vessel map of the retinal oximetry image (RO) shows color-coded oxygen saturation (SO_2_) values of retinal vessels within the peripapillary annulus of a glaucoma patient (right eye). We evaluated the average SO_2_ parameters (A-SO_2_, V-SO_2_ and A-V SO_2_; %).

**Figure 2:**
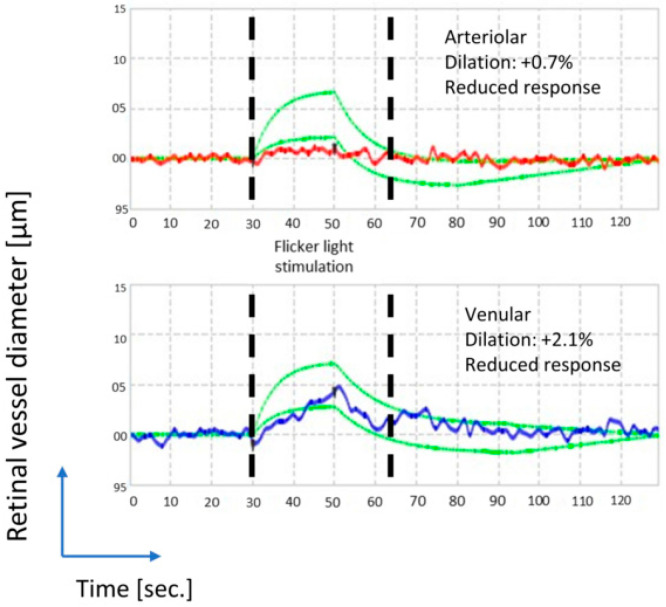
Represents an example of vessel responsiveness measurements of a glaucoma patient to FL, using a retinal vessel analyser (RVA). On the *y*-axis the diameter (µm) of the selected vessel is plotted, and on the *x*-axis, the measurement during one period of flicker stimulation (sec). Retinal vessel responses to FL in the preselected vessel parts in contraction and in dilatation (%) were evaluated. Labelled in red (on the top) is the arterial and in blue (on the bottom), the venular diameter variation. The dashed green lines label the normal range. Both the AFR and the VFR are attenuated in glaucoma eyes, as exemplified here.

Both retinal vessel oximetry (RO) imaging and retinal vascular responsiveness (RVR) to flicker light (FL) were obtained during one visit, with 20 min break between the tests (Figure 1 and Figure 2). In order to avoid the effect of flickering light on retinal vessel diameter and oxygen saturation, as reported recently [27,28,62], the RO was performed before measuring the RVR to flickering light. Following pupil dilatation, RO was performed on both eyes of our patients, starting with the right eye [63]. To stabilize hemodynamic conditions, before RO imaging and retinal flicker light responses were recorded, patients were seated for 5–10 min. In addition, patients were instructed to avoid caffeine and nicotine consumption starting the day before the RO/RVA examination day [64]. 

### 4.4. Retinal Vessel Oximetry Imaging

Retinal vessel oximetry imaging (RO) was performed using the oximetry unit of a retinal vessel analyser (RVA: Imedos UG, Jena, Germany). Fundus images were taken within 50° of the posterior pole of the retina using a fundus camera FF450 (Carl Zeiss Meditec, Jena, Germany), coupled to the charge-coupled device chip (CCD). The main principle of the spectrophotometric retinal oximetry is comparing two retinal images simultaneously. One image was taken with an oxygen non-sensitive filter at 548 ± 10 nm wavelength, and a second image was taken with an oxygen-sensitive filter at 610 ± 10 nm wavelength. The software (Vesselmap) then calculated the optical density ratio (ODR) of the two images and, thus, the mean oxygen saturation of the evaluated retinal vessel. The data were presented as colour-coded oxygen saturation graphics (Figure 1). An example of oximetry reading of a glaucoma patient is shown in Figure 1. Patients and controls were adapted for 10 min to mesopic conditions, as described before [63]. Images of both eyes were obtained, starting with the right eye. The oxygen saturation was measured in all major retinal arterioles (A-SO_2_) and venules (V-SO_2_) within a distance of 0.5–1.0 optic disc diameter from the optic disc edge. We computed also the arterio–venular difference (A-V SO_2_), known to be proportional to the oxygen use. In addition, we calculated the mean peripapillary oxygen exposure [ppO_2_E; %/µm] by dividing the mean A-V SO_2_ by the mean retinal nerve fibre layer (RNFL) thickness. 

### 4.5. Retinal Vessel Analyser

The retinal vessel analyser (RVA) consists of a fundus camera (Mydriatic), a digital high-resolution CCD video camera system (charge-coupled device) and software [33]. The vessel tracking system allows the online and offline analyses of the retinal vessel of interest: the vessel segment is tracked continuously, where blinking artefacts and bad quality recording periods are ignored, thus providing a clear analytical and graphical results. Applying flicker light (FL) stimulation (frequency: 12.5 Hz), provided by optoelectronic shutter of RVA, the retinal tissue can be artificially stimulated. 

In our study, depending on the pathology, one or both eyes of each glaucoma patient were exposed for 50 s to a baseline constant illumination. Afterwards, three periods of 12.5 Hz flicker stimulation (each of 20 s duration, interrupted by 80 s of constant illumination) were performed. Retinal vessel responses to FL in the preselected vessel parts in dilatation were recorded and the respective change in vessel dilatation from baseline was calculated (as percentage, %). Instead of dividing our glaucoma patients based on their history of “vasospastic dysregulation”, we subdivided them objectively based on their vasospastic reactions, taking into account the median VFR and AFR dilatation from the baseline diameter, as described below. Eligibility criteria for RVA vessel selection were as follows: good contrast between vessels and surrounding retinal tissue, a randomly selected superior or inferior temporal vessel, and measurements done within 1 to 2 disc diameters from the optic disc margin. 

### 4.6. Statistical Analysis

For the statistical analysis of the data, a linear mixed-effects model, was applied. Mixed-effects models are suitable for repeated measurements data, also allowing an independent evaluation of each eye of the same subject. Oxygen saturation values (A-SO_2_, V-SO_2_ and A-V SO_2_, %) were compared between glaucoma patients and controls. *p*-values ≤ 0.05 were considered statistically significant. 

Relationships between the metabolic alteration and the extent of glaucomatous damage within the glaucoma group were analysed further as follows:

1. According to the median retinal vascular responsiveness to flicker light, glaucoma subjects were grouped as follows:
  - By median arteriolar flicker responsiveness (1.80%; AFR) in:
    - AFR ≤ 1.80% group: indicates ≤1.80% dilatation from the baseline diameter;
    - AFR ≤ 1.80% group: indicates >1.80% dilatation from the baseline diameter.
  - By median venular flicker responsiveness (2.90%; VFR) in:
    - VFR ≤ 2.90% group: indicates ≤2.90% dilatation from the baseline diameter;
    - VFR > 2.90% group: indicates >2.90% dilatation from the baseline diameter.
2. Furthermore, we tested a hypothesis that the responsiveness to FL stimulation depends on the structural and functional alterations in glaucoma patients and sought to find out whether this relationship is different between the vascular responsiveness glaucoma groups (AFR, VFR).
3. In addition, in order to obtain the mean peripapillary oxygen exposure per micron of nerve fibre layer thickness, ppO_2_E [%/µm], the mean A-V SO_2_ was divided by the mean RNFL thickness. Glaucoma patients were divided depending on the ppO_2_E values into two groups as follows:
  - ppO_2_E ≤ 0.46 group: indicates ppO_2_E values ≤ 0.46%/µm;
  - ppO_2_E > 0.46 group: indicates ppO_2_E values > 0.46%/µm.
4. We studied then the relationships between oxygen saturation, flicker responsiveness and ppO_2_E within the glaucoma group.

In the present study, in order to predict the effect of oximetry alterations on vascular responsiveness measurements, the eye- and the group-effect were taken into account in our ANOVA-based mixed-effects model, where the eye and the group were treated as fixed factors and the subject as a random factor. 

## 5. Conclusions

In conclusion, based on results of the present study, we were able to document the link between the presence of vascular dysregulation and increased peripapillary oxygen exposure in eyes with glaucoma. 

The novelty of the study is that reduced venular responsiveness to flicker light is associated with increased peripapillary oxygen exposure. 

We hypothesize that ganglion cell and their axons in glaucomatous eyes with reduced retinal vascular responsiveness are prone to be more exposed to higher oxidative stress, probably contributing to the further progression of glaucomatous damage.

Thus, peripapillary oxygen exposure (ppO_2_E) may serve as a non-invasive indicator for increased oxidative stress in eyes with glaucoma and be defined as a risk factor for disease progression in glaucoma patients who also display vascular dysregulation.

However, some limitations are to be considered in further studies, for instance, the influence of gender, blood pressure and pulse rate on retinal vessel oxygen saturation, as well as the influence of anti-glaucoma medication and surgery on peripapillary oxygen exposure in glaucoma. 

Additionally, in terms of calculating the peripapillary oxygen exposure as a parameter, a proper RNFL measurement is required. Furthermore, a clear media and a good image quality are mandatory in order to obtain reliable retinal oximetry, flicker responsiveness and SD-OCT measurements, where the acquisition, for instance in elderly glaucoma patients, may involve some difficulties.

Last but not at least, care should be taken in the interpretation of the ppO_2_E as a parameter for oxygen consumption not only in RGCs but probably in the entire inner retina.

## Figures and Tables

**Table 1:** Oxygen saturation values (%) and peripapillary oxygen exposure values (ppO_2_E; %/μm) in control and glaucoma patients. Statistically significant values (*p* < 0.05) are given in bold.

Parameters	Groups	Nr. Eyes	Mean	SD	95% CI		*p*-Values between Control and Glaucoma Patients
					Lower Limit	Upper Limit	
A–SO_2_ (%)	Controls Glaucoma	21	93.44	4.97	91.58	95.31	**0.016**
		46	96.18	3.64	94.98	97.38	
V–SO_2_ (%)	Controls Glaucoma	21	54.21	9.10	52.97	57.44	**<0.001**
		46	61.66	6.04	59.58	63.73	
A–V SO_2_ (%)	Controls Glaucoma	21	39.25	7.96	36.45	42.06	**0.011**
		46	34.86	6.04	33.06	36.67	
ppO_2_E (%/µm)	Controls Glaucoma	21	0.40	0.09	0.34	0.44	**0.024**
		46	0.46	0.10	0.43	0.49	

**Table 2:** Oxygen saturation and peripapillary oxygen exposure values in glaucoma patients in groups. Based on the median VFR glaucoma subjects were divided in two groups: VFR group with ≤2.9% VFR dilatation from the baseline diameter and the VFR group with >2.9% VFR dilatation from the baseline diameter. The respective oxygen saturation and peripapillary oxygen exposure values of controls are given for comparison. Statistically significant values (*p* < 0.05) are given in bold.

Parameters	Control vs. Glaucoma Groups by Median VFR	Nr. Eyes	Mean	SD	95% CI		*p*-Values between Glaucoma Groups
					Lower Limit	Upper Limit	
A–SO_2_ (%)	Controls	46	93.44	4.97	91.58	95.31	
	VFR ≤ 2.9%	27	96.50	4.39	93.85	99.16	0.517
	VFR > 2.9%	19	97.38	3.03	95.63	99.13	
V–SO_2_ (%)	Controls	46	5.21	9.10	52.97	57.44	
	VFR ≤ 2.9%	27	62.90	6.23	59.13	66.66	0.391
	VFR > 2.9%	19	60.72	6.28	57.09	64.34	
A–V SO_2_ (%)	Controls	46	39.25	7.96	36.45	42.06	
	VFR ≤ 2.9%	27	34.64	4.51	31.92	37.37	0.294
	VFR > 2.9%	19	36.68	5.28	33.64	39.72	
ppO_2_E (%/µm)	Controls	21	0.40	0.09	0.34	0.44	
	VFR ≤ 2.9%	27	0.49	0.08	0.44	0.54	**0.027**
	VFR > 2.9%	19	0.43	0.06	0.40	0.47	

**Table 3:** Correlations (*p*-values) between retinal vessel oxygen saturation and peripapillary oxygen exposure with the extent of glaucomatous damage. *p*-values < 0.05 are considered statistically significant and are given in bold.

Retinal Vessel Oxygen Saturation and Peripapillary Exposure	Nr. Eyes	Neuroretinal Rim Area (mm^2^) *p*-Values	RNFL Thickness (µm) *p*-Values	Visual Field (MD, dB) *p*-Values
A–SO_2_ (%)	46	0.236	0.297	0.392
V–SO_2_ (%)	46	**0.012**	**0.001**	**0.002**
A-V SO_2_ (%)	46	**0.007**	**<0.001**	**0.001**
ppO_2_E (%/µm)	46	**<0.001**	**0.017**	**0.012**

**Table 4:** Structural and functional glaucomatous damage subdivided in groups by median venular flicker vascular responsiveness: VFR group with ≤2.9% VFR dilatation from the baseline diameter and VFR group with >2.9% VFR dilatation from the baseline diameter. *p*-values < 0.05 are considered statistically significant and are given in bold.

Glaucomatous Damage	Groups by Median VFR	Nr. Eyes	Mean	SD	95% CI		*p*-Values between Glaucoma VFR Groups
					Lower Limit	Upper Limit	
Neuroretinal rim area (mm^2^)	VFR ≤ 2.9%	27	0.78	0.32	0.98	0.29	0.098
	VFR > 2.9%	19	0.95	0.21	1.08	0.60	
RNFL thickness (µm)	VFR ≤ 2.9%	27	72.23	12.09	64.92	79.54	**0.014**
	VFR > 2.9%	19	85.31	13.67	77.42	93.21	
Visual field (MD, dB)	VFR ≤ 2.9%	27	4.73	3.66	2.52	6.94	0.270
	VFR > 2.9%	19	3.49	5.29	0.44	6.55	

**Table 5:** Peripapillary oxygen exposure (ppO_2_E; %/μm) of controls against both glaucoma groups, where the glaucoma group is divided by oxygen consumption into ppO_2_E group with ≤46%/μm and ppO_2_E group with >46%/μm. Statistically significant values are given in bold and those showing a trend: in italics underlined.

Parameters	Glaucoma Groups by Median ppO_2_E	Nr. Eyes	Mean	SD	95% CI		*p*-Values between Subgroups: Controls: ≤0.46 Controls: >0.46 ≤0.46: >0.46
					Lower Limit	Upper Limit	
A–SO_2_ (%)	Controls	21	93.44	4.97	91.58	95.31	0.184
	ppO2E ≤ 0.46	25	95.43	3.73	93.80	97.07	*0.065*
	ppO2E > 0.46	21	96.26	3.76	98.08	98.08	0.503
V–SO_2_ (%)	Controls	21	54.21	9.10	52.97	57.44	**0.015**
	ppO2E ≤ 0.46	25	62.29	5.67	59.33	65.25	0.526
	ppO2E > 0.46	21	57.92	8.86	54.63	61.21	*0.053*
A–V SO_2_ (%)	Controls	21	39.25	7.96	36.45	42.06	*0.060*
	ppO2E ≤ 0.46	25	33.59	5.24	31.16	36.01	0.565
	ppO2E > 0.46	21	38.54	6.48	35.84	41.24	**0.008**

## Data Availability

The data presented in this study are available on request from the corresponding author. The data will be accessible only to authorized staff for scientific purposes.
